# CTC-mRNA (AR-V7) Analysis from Blood Samples—Impact of Blood Collection Tube and Storage Time

**DOI:** 10.3390/ijms18051047

**Published:** 2017-05-12

**Authors:** Alison W. S. Luk, Yafeng Ma, Pei N. Ding, Francis P. Young, Wei Chua, Bavanthi Balakrishnar, Daniel T. Dransfield, Paul de Souza, Therese M. Becker

**Affiliations:** 1 Centre for Circulating Tumour Cell Diagnostics and Research, Ingham Institute for Applied Medical Research, 1 Campbell St., Liverpool, NSW 2170, Australia; alisonluk@gmail.com (A.W.S.L.); yafeng.ma@unsw.edu.au (Y.M.); Pei.Ding@sswahs.nsw.gov.au (P.N.D.); francis.young@student.unsw.edu.au (F.P.Y.); P.DeSouza@westernsydney.ed u.au (P.d.S.); 2 Department of Medical Oncology, Liverpool Hospital, Elizabeth St & Goulburn St, Liverpool, NSW 2170, Australia; Wei.Chua2@sswahs.nsw.gov.au (W.C.); Bavanthi.Balakrishnar@sswahs.nsw.gov.au (B.B.); 3 Western Sydney University Clinical School, Elizabeth St, Liverpool, NSW 2170, Australia; 4 South Western Clinical School, University of New South Wales, Goulburn St., Liverpool, NSW 2170, Australia; 5 Tokai Pharmaceuticals, Inc., 255 State Street, 6th Floor, Boston, MA 02109, USA; dan@siamab.com

**Keywords:** circulating tumour cell, biomarker, androgen receptor, AR-V7, droplet digital polymerase chain reaction (ddPCR), blood storage tube

## Abstract

Circulating tumour cells (CTCs) are an emerging resource for monitoring cancer biomarkers. New technologies for CTC isolation and biomarker detection are increasingly sensitive, however, the ideal blood storage conditions to preserve CTC-specific mRNA biomarkers remains undetermined. Here we tested the preservation of tumour cells and CTC-mRNA over time in common anticoagulant ethylene-diamine-tetra-acetic acid (EDTA) and acid citrate dextrose solution B (Citrate) blood tubes compared to preservative-containing blood tubes. Blood samples spiked with prostate cancer cells were processed after 0, 24, 30, and 48 h storage at room temperature. The tumour cell isolation efficiency and the mRNA levels of the prostate cancer biomarkers androgen receptor variant 7 (AR-V7) and total AR, as well as epithelial cell adhesion molecule (EpCAM) were measured. Spiked cells were recovered across all storage tube types and times. Surprisingly, tumour mRNA biomarkers were readily detectable after 48 h storage in EDTA and Citrate tubes, but not in preservative-containing tubes. Notably, AR-V7 expression was detected in prostate cancer patient blood samples after 48 h storage in EDTA tubes at room temperature. This important finding presents opportunities for measuring AR-V7 expression from clinical trial patient samples processed within 48 h—a much more feasible timeframe compared to previous recommendations.

## 1. Introduction

Circulating tumour cells (CTCs) are cells shed from tumours into the peripheral blood, and are believed to be the mechanism for metastasis [1,2]. The enumeration of CTCs has great prognostic value, and further molecular profiling of CTCs has great potential in providing insights on cancer progression, identifying CTC specific molecular therapeutic targets, determining prognostic and relapse indicators, and allowing longitudinal monitoring of disease response to treatments [3,4]. Therefore, relatively simple, non-invasive CTC analysis has exciting potential to investigate changes in tumour biomarkers during a cancer patient’s disease progression. In prostate cancer, CTC counts have been associated with prognosis and importantly, isolated CTCs can function as a surrogate tumour samples to detect therapy-determining biomarkers, including the mRNA based androgen receptor variant AR-V7 in CTCs [5,6,7,8]. However, CTC analysis is still a rapidly-evolving field due to the complexities of isolation and detection of true CTCs. While it is estimated that 10^6^ CTCs are shed per 1 g of tumour tissue per day, CTCs are thought to have a short half-life of less than 3 h in the bloodstream [9,10,11,12]. Hence, a major technical challenge for CTC analysis has been efficient recovery of rare CTCs from a background of approximately 10^9^ erythrocytes and 10^7^ leukocytes per mL of blood, and this has spurred the development of improved technologies for CTC isolation [13,14,15]. Despite these advances, feasible CTC analysis for clinical trials involving multiple sites are particularly challenging, due to strict requirements for pre-analytical conditions of blood samples in transport and storage, and time restrictions to ensure CTC integrity and biomarker detectability is maintained. Parameters, such as blood tube composition, storage or shipping temperature, sample agitation, and delays in sample processing in a large-throughput laboratory need to be considered before such measurements are conducted for clinical trials. Ideally, CTCs should be protected from apoptosis or cell lysis and, importantly, preserve relevant tumour biomarkers, an issue that is considered especially challenging for mRNA-based biomarkers.

Ethylene-diamine-tetra-acetic acid (EDTA) and acid citrate dextrose solution B (Citrate) are commonly used as anticoagulants in blood tubes for pathology blood tests. These tubes are compatible with downstream polymerase chain reaction (PCR) analysis [16,17] and have also been used for CTC isolation. However, as these tubes do not contain fixatives, early studies recommended that blood should be processed within 24 h, and ideally within 5 h, as it was thought that CTCs rapidly enter apoptosis [18]. To target this problem, new blood tubes have been designed with preservatives intended to extend time before sample processing. Cell-free DNA blood collection tubes (DNA BCT) and Cell-free RNA blood collection tubes (RNA BCT) (Streck, Omaha, NE, USA) are proposed to stabilise nucleated blood cells, preventing the release of cellular DNA and RNA into the plasma, and also inhibiting degradation of cell-free DNA and RNA [19,20]. Therefore, these tubes might be advantageous for CTC sample analysis. Additionally, Cyto-Chex blood collection tubes (Cyto-Chex BCT) were originally designed to preserve cell surface antigens for white blood cell immunophenotyping [21] which may allow improved CTC recovery by immunomagnetic isolation.

There have been previous reports suggesting the added preservatives in BCTs indeed aids CTC stability [22,23]. However, the preservative effects on actionable mRNA-based tumour biomarkers, such as AR-V7 has, to our knowledge, not been tested thoroughly in CTCs isolated from any blood collection tube type. We have previously reported a very sensitive method to detect AR-V7, an emerging RNA-based prostate cancer biomarker in prostate cancer patient CTC samples, showing that AR-V7 is not expressed in residual blood cells, and is expressed heterogeneously in CTCs [7]. In this study, we compare the effects of preservative-containing BCTs to commonly used EDTA and Citrate blood tubes on tumour cell isolation and detection of AR-V7 using our sensitive method.

## 2. Results

### 2.1. Spiked Cell Recovery

**Figure 1:**
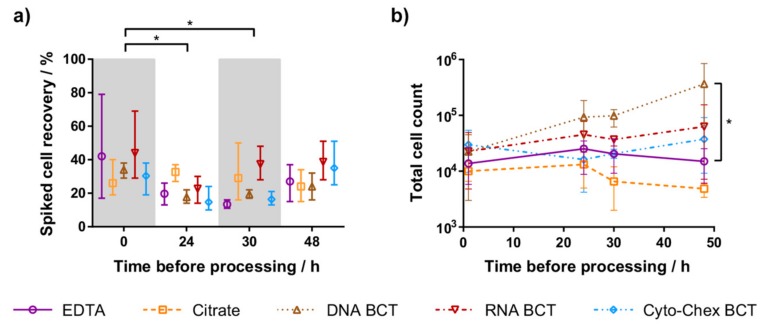
(**a**) Recovery of spiked 22Rv1 cells after tumour cell enrichment. The mean recovery at 24 h and 30 h was significantly different from recovery at 0 h (* *p* < 0.05). (**b**) Total cell count (recovered spiked cells and residual leukocytes after cell enrichment) from samples processed 0 h, 24 h, 30 h, and 48 h after spiking. At 48 h, DNA blood collection tubes (DNA BCT) cell count was significantly different from ethylene-diamine-tetra-acetic acid (EDTA) (* *p* < 0.05). For both (**a**,**b**), symbols represent the mean from three independent experiments, and whiskers represent the range.

To test CTC preservation and recovery from the five different blood tubes, CTCs were modelled by spiking defined cell numbers of the 22Rv1 prostate cancer cell line into healthy female donor blood. At 0, 24, 30, and 48 h after spiking, tumour cells were enriched and enumerated. Three experiments were performed using 192 mL blood each from three healthy donors. The mean recovery of spiked cells ranged from 13% to 44% across all time points. All blood tubes showed preservation of spiked cells up to 48 h, with mean recovery between 24% and 39% (Figure 1a). The mean recovery of all blood tubes at each time point was compared against recovery at 0 h. Processing samples at 24 and 30 h resulted in significantly lower recovery than when processed at 0 h (*p* < 0.05). Within each time point, the recovery of each blood tube was compared to EDTA. No blood tube had significantly different recovery from EDTA, but the mean recovery of RNA BCT exceeded EDTA at each time point (*p* < 0.05).

### 2.2. Leukocyte Contamination

DNA BCT and RNA BCT generally had increased total cell counts (tumour cells plus residual co-purified leucocytes) when samples were processed later (Figure 1b). In comparison, EDTA, Citrate and Cyto-Chex BCT cell counts remained similar across all time points. When compared to EDTA tubes, DNA BCT and RNA BCT had higher mean cell counts at each time point, whereas Citrate tubes gave lower cell counts. A two-way ANOVA of each blood tube compared to EDTA at the same time point indicated that for samples processed at 48 h, DNA BCT total cell counts were significantly higher than for EDTA tubes (*p* < 0.05).

### 2.3. Cellular RNA Recovery

**Figure 2:**
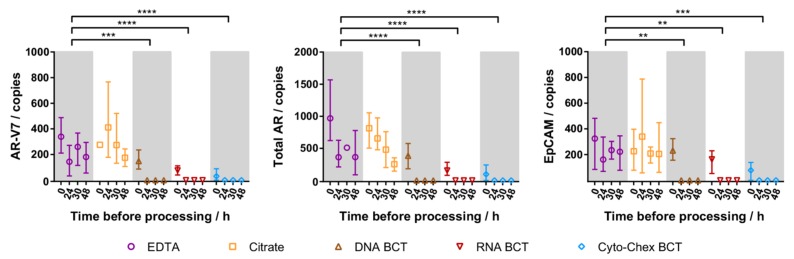
Expression of spiked tumour cell specific genes in samples processed 0 h, 24 h, 30 h, and 48 h after spiking. Symbols represent mean expression from three independent experiments, whiskers represent the range, and are not shown when smaller than the data symbol. The mean gene expression in DNA BCT, RNA BCT, and Cyto-Chex BCT were significantly different from EDTA (** *p* < 0.01, *** *p* < 0.001, **** *p* < 0.0001). AR-V7: androgen receptor variant 7; AR: androgen receptor.

To evaluate the ability of each blood tube in preserving cellular RNA, tumour cell-specific gene expression (AR-V7, total AR and epithelial cell adhesion molecule (EpCAM)) was measured by droplet digital PCR (ddPCR) for each blood tube at each tumour cell enrichment time point (Figure 2). Cells from EDTA and Citrate tubes generally showed decreased mRNA detection the longer the sample storage time, with mRNA biomarkers still readily detectable even after 48 h. In all BCT samples, gene expression was low when processed immediately and undetectable after any storage duration. Thus, while mRNA detection from Citrate and EDTA blood tube samples was similar, BCT samples showed a striking loss in detectable gene expression compared to EDTA tube samples (*p* < 0.01).

### 2.4. Increased Proteinase K Treatment

**Figure 3:**
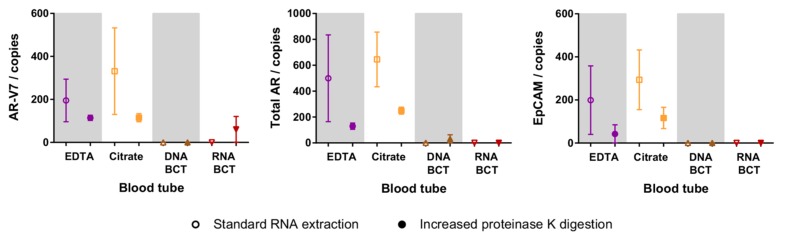
Effect of increased proteinase K treatment on the detection of spiked cell specific genes in samples processed 48 h after spiking. Symbols represent the mean expression from two independent experiments, whiskers represent the range, and are not shown when smaller than the data symbol. EpCAM: epithelial cell adhesion molecule.

We also investigated the effect of increased proteinase K digestion on the cellular RNA recovery as per manufacturer’s suggestions for the BCTs. 22Rv1 cells were spiked into a new set of blood tubes (EDTA, Citrate, DNA BCT, and RNA BCT), followed by enrichment after 48 h of storage. RNA was extracted with and without additional 2 h proteinase K treatment, and gene expression was measured in RNA samples from two independent experiments. In EDTA and Citrate blood tubes, increased proteinase K digestion did not aid RNA recovery but decreased the number of measured copies of all three genes (Figure 3). In DNA BCT and RNA BCT, there was no detectable AR-V7, total AR or EpCAM with standard RNA extraction, and with increased digestion there was a small, but statistically insignificant, increase in the detection of total AR and AR-V7 in DNA BCT and RNA BCT, respectively.

### 2.5. Patient CTC Cellular RNA Detection

**Figure 4:**
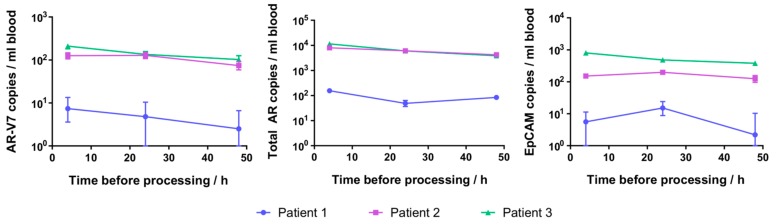
Detection of gene expression in prostate cancer patient circulating tumour cells (CTCs) isolated 4, 24, and 48 h after blood collection in EDTA tubes. Error bars represent 95% confidence intervals of droplet digital polymerase chain reaction (ddPCR) measurements, and error bars smaller than the data symbols are not shown.

Given that spiked cell-specific gene expression was detectable even in samples processed 48 h following spiking of 22Rv1 cells into fresh blood in EDTA and Citrate tubes, we wished to confirm that AR-V7 also remains detectable that long in patient-derived CTCs. Common EDTA tube blood samples from three prostate cancer patients were processed at 4, 24, and 48 h after collection. After CTC enrichment, all patient samples had detectable AR-V7, total AR and EpCAM at all time points (Figure 4). AR-V7 expression varied between the three patients, while being comparable to the expression detected for these patients in a sample collected previously (Table 1). The AR-V7 and total AR detected in patient samples decreased with longer time before processing, but the decrease was not statistically significant.

## 3. Discussion

### 3.1. Tumour Cell Preservation

New blood tubes such as DNA BCT, RNA BCT, and Cyto-Chex BCT contain formaldehyde-free fixatives in addition to traditionally used anticoagulants in blood tubes [19,20,21]. As these BCTs were previously shown to preserve leukocytes and prevent the release of cellular RNA into plasma, even after over three days of storage at room temperature [20,24,25], our study compared commonly-used EDTA and Citrate tubes with BCTs in terms of the ability to preserve modelled CTCs (spiked cultured tumour cells) and of greater interest, cellular RNA.

From our study, the total cell count by Hoechst staining showed that leukocyte retention after tumour cell enrichment increased over time in DNA BCT compared to other blood tubes. Since BCTs were designed for analysis of plasma cell-free nucleic acids, this has not previously been a concern. However, downstream analysis of CTCs can be adversely affected by contaminating leukocytes, such as for single CTC isolation or CTC nucleic acid analysis. Disregarding background leukocytes, intact tumour cells were visible under bright field microscopy from all blood tubes after 48 h (data not shown), and enumeration of cell tracker-stained cells indicated that the recovery of spiked tumour cells was not significantly different across all blood tube types within each time point. Furthermore, although spiked cell recovery generally decreased with longer storage time before processing, there was no significant difference between recovery at 0 and 48 h, with a mean recovery of 35% and 30%, respectively. Our results are in agreement with a previous study which found that CTC yields from lung cancer patients did not decline significantly when processed after 24, 48, or 72 h storage in EDTA tubes [26]. We suggest that the observed variation in spiked cell recovery in our data can be mostly attributed to the difficulties of spiking exact cell numbers of the 22Rv1 cell line, which shows a very strong tendency towards cell clustering. Nevertheless, this line was chosen due to its high AR-V7 expression, and cell strainers were used to keep the effects of cell aggregates to a minimum. Conversely, our results differ from a recent study which reported that the recovery of 2000 spiked MCF-7 breast cancer cells from DNA BCT after one day and four days was very similar at 60% and 58%, respectively, while the recovery decreased from 32% to 16% for EDTA tubes [23]. The discrepancies between the results may be attributed to the difference in the spiked cell line, spiked cell numbers, method of tumour cell isolation, and detection and blood storage time. Importantly, the observed differences in the limited number of studies in this area highlights the need for more research to further compare and improve methods of CTC isolation after blood storage.

Despite common recommendations to process blood samples as soon as possible to reduce cell lysis, our data indicates that cell fixation is not necessary for the recovery of tumour cells, and common EDTA or Citrate tubes are sufficient for tumour cell detection within 48 h.

### 3.2. Cellular RNA Preservation

Analysis of gene expression from CTCs can provide valuable information on CTC activity and cancer progression, but is particularly challenging as delays in blood sample processing may cause alterations in CTC gene expression, and existing mRNAs may have a short half-life. DNA BCT and RNA BCT were designed to preserve cellular DNA and RNA, respectively, from being released and, hence, were expected to perform better than commonly-used EDTA and Citrate tubes, which only contain anticoagulants. We used our previously-reported, highly-sensitive and specific ddPCR assay to screen for AR-V7, total AR, and EpCAM; this assay has shown before that AR-V7 and total AR were highly expressed in the 22Rv1 cell line or patient CTCs while having no, or negligible, expression in healthy control peripheral lymphocytes [7]. We were surprised to find AR-V7, total AR, and EpCAM readily detectable after 48 h in common EDTA and Citrate tubes. The data suggest that mRNAs encoding these genes have relatively long half-life and/or are continuously expressed by viable tumour cells during storage in a blood sample. Additionally, the detection of mRNA confirms that cells remain intact in the blood sample as it is well established that any released free RNA will be quickly degraded by RNases in the blood [27]. Since cultured 22Rv1 cells are potentially more robust and survive extended storage in blood samples better than CTCs, we confirmed that AR-V7 detection after 48 h storage was translatable to patient CTC samples by testing three patients previously shown to have high CTC AR-V7 expression [7]. Although a small decrease in AR-V7 levels after 48 h was detected in these patient samples, the decrease was marginal in this time frame and even in patient blood with low AR-V7, CTC derived AR-V7 was still detectable after 48 h blood storage at room temperature. AR-V7 expression was comparable to the previous testing of the same patients [7].

In contrast to our data from blood storage in common blood tubes, the BCT samples processed immediately produced much lower detectable tumour cell specific mRNA, and any storage of spiked blood samples in these tubes prevented detection of gene expression completely. We propose that the preservative in the tubes renders RNA inaccessible, likely due to RNA cross-linking with proteins and DNA, which would be incomplete when samples are processed immediately, explaining the limited gene expression detected for our 0 h samples. This interpretation is supported by a report that spiked breast cancer cells could be retrieved from DNA BCTs after up to 72 h storage and had accessible DNA, however, whole genome amplification (WGA) yielded consistently significantly less DNA than WGA from EDTA tubes [22]. Alternatively, RNA could have degraded in the BCTs in our study, however, mRNA was reported detectable by in situ hybridisation from spiked tumour cells isolated from blood stored for four days at room temperature in DNA BCTs [23], suggesting that RNA remains intact but possibly cross-linked, which would interfere less with in situ hybridisation than RNA extraction. While, according to the manufacturers guidelines, RNA should be extractable from samples stored in BCTs, an extended protein K digest is recommended, which might help to reverse cross-linking effects. We, therefore, investigated whether increased proteinase K treatment during RNA extraction would allow relevant gene expression detection for samples stored for 48 h. However, increased proteinase K digest failed to produce relevant effects and decreased RNA detectability in EDTA and Citrate tubes.

Previous studies showed BCTs to preserve leukocyte cellular RNA, and prevent cellular RNA contamination of cell-free RNA [20,24]. Additionally, the detection of cellular GAPDH, c-Fos and p53 RNA was reported to show variation in expression in neutrophils from blood samples stored over three days in EDTA tubes, whereas there was no change when stored in RNA BCT [25]. While the reasons for the difference to our data are not completely clear, it stands out that all genes tested in that study are abundantly-expressed genes in common blood cells, which might be easier to detect. We have not extended testing past 48 h, as blood samples from within Australia can be processed in this time frame in our facility; however, it is an area of interest for future studies to determine the maximum storage time for EDTA and Citrate tubes.

From our data, it is evident that CTC-derived AR-V7 can be detected from blood stored in commonly-used EDTA and Citrate tubes for up to 48 h, while blood tubes with preservatives (DNA BCT, RNA BCT, and Cyto-Chex BCT) should not be used for CTC isolation if downstream RNA analysis by PCR is intended.

## 4. Materials and Methods

### 4.1. Blood Collection

Prostate cancer patients (*n* = 3) and healthy female blood donors (*n* = 4) provided written informed consent to participate in the study. The study was approved by the South Western Sydney Local Healthy District Ethics Committee, Australia (HREC/13/LPOOL/158; 02/09/2013). Peripheral blood was drawn by venipuncture into blood tubes with the initial 3 mL discarded to prevent keratinocyte contamination and false positive CTCs being present. For the main comparison experiment, a total of 192 mL blood from each healthy donor (*n* = 3) was drawn into a total of 24 blood tubes including: 4 × 9 mL K_3_EDTA tube (Greiner Bio-One, Kremsmünster, Austria), 4 × 9 mL acid citrate dextrose-B tube (Greiner Bio-One, Kremsmünster, Austria), 4 × 10 mL Cell-free DNA BCT (Streck, Omaha, NE, USA), 4 × 10 mL Cell-free RNA BCT (Streck, Omaha, NE, USA), and 4 × 2 × 5 mL Cyto-Chex BCT (Streck, Omaha, NE, USA). In the follow-up experiment to test increased proteinase K treatment, 38 mL blood from two healthy donors was drawn into one set of EDTA, Citrate, and DNA BCT and RNA BCT tubes. In the experiment with prostate cancer patients, blood was drawn into 3 × 6 mL K_2_EDTA tubes (BD, Franklin Lakes, NJ, USA) available in the clinic.

### 4.2. Cell Spiking

The human prostate cancer cell line 22Rv1 was purchased from the American Type Culture Collection (In Vitro Technologies, Melbourne, Australia). Cells were routinely passaged in Roswell Park Memorial Institute culture medium (RPMI 1640) (Lonza, Basel, Switzerland) supplemented with 10% fetal bovine serum (FBS) (Invitrogen, Carlsbad, CA, USA) in a humidified incubator with 5% CO_2_ at 37 °C. For spiking experiments, 22Rv1 cells, cultured for two days after passaging, were gently detached with accutase (Sigma-Aldrich, St. Louis, MO, USA), washed with phosphate buffered saline (PBS), and passed through a 20 µm pre-separation filter “cell strainer” (Miltenyi Biotec, Bergisch Gladbach, Germany) to remove cell aggregates. Cells were then incubated with 15 µM CellTracker Green CMFDA (Life Technologies, Carlsbad, CA, USA) in 100 µL serum-free RPMI media at 37 °C for 1 h. Stained 22Rv1 cells were washed with PBS and a known number (100–200 cells) were spiked into blood tubes. The spiked input cell numbers were verified by aliquoting the same volume onto glass slides in between inoculating blood samples, Hoechst staining, and enumeration by fluorescent microscopy.

### 4.3. Tumour Cell Enrichment from Whole Blood

Spiked blood samples were enriched for tumour cells after storage (dark, room temperature) for 0, 24, 30, and 48 h. The peripheral blood mononuclear cell (PBMC) layer was extracted using 50 mL SepMate tubes and Lymphoprep according to the manufacturer’s instructions (Stemcell Technologies, Vancouver, BC, Canada). PBMCs were washed with separation buffer (PBS with 0.5% FBS and 2 mM EDTA), then incubated at 4 °C for 30 min with 50 µL FcR blocking reagent (Miltenyi Biotec, Bergisch Gladbach, Germany), 50 µL EpCAM conjugated immunomagnetic microbeads (Miltenyi Biotec, Bergisch Gladbach, Germany), 1 µL 50× Hoechst (Fluxion, San Francisco, CA, USA), and made up to a total volume of 500 µL with separation buffer. Cell separation was performed with the Posselds program on the AutoMACS Pro Separator (Miltenyi Biotec, Bergisch Gladbach, Germany). After separation, the positive selected fraction was separated into two aliquots: one was kept on ice until enumeration on the same day, while the other was centrifuged at 400× *g* for 10 min, and the pellet frozen at −80 °C until RNA extraction. For testing of extended proteinase K treatment, samples were enriched after 48 h, with both aliquots frozen at −80 °C until RNA extraction. For prostate cancer patient samples, one blood tube from each patient was processed within 4 h of blood draw, and the other two blood tubes were processed after 24 and 48 h of storage, respectively, before freezing at −80 °C until RNA extraction. Supplementary Figure S1 illustrates the different work flows.

### 4.4. Cell Enumeration

The enriched enumeration sample was mounted onto slides coated with 2% bovine serum albumine (BSA), then visualised and scanned at 20× magnification with a CellCelector microscope (ALS GmbH, Jena, Thüringen, Germany). The exposure times for the instrument’s DAPI and FITC channels were 50 ms and 100 ms, respectively. Scanned images were analysed with ALS CellCelector software v3.0 (ALS GmbH). Nucleated (Hoechst-positive) cells were detected for total cell counts, while cells positive for both Hoechst and CellTracker were considered recovered spiked cells.

To calculate the percentage recovery of spiked cells, the number of cells enumerated after tumour cell enrichment was multiplied by two (to account for CTCs being enumerated in half of the sample while the other half was processed for RNA) and then divided by the original number of spiked cells as determined from input controls.

### 4.5. Cellular RNA Extraction

Total RNA was extracted from the second enriched sample or from CTC-enriched patient samples with the Total RNA Purification Micro Kit (Norgen Biotek Corp., Thorold, ON, Canada). RNA was double-eluted in 20 µL followed by 10 µL molecular-grade H_2_O. For complementary DNA (cDNA) synthesis, 15 µL of eluted RNA was added to form a total volume of 20 µL with the SensiFAST cDNA Synthesis Kit (Bioline, London, UK). For testing increased proteinase K digestion, 0.5 mg of DNAase and RNAase free proteinase K (Bioline) was added with buffer RL at the cell lysate preparation step, and the sample was incubated for 2 h at 60 °C before the addition of ethanol.

### 4.6. Digital Droplet PCR

Quantification by ddPCR was performed for three tumour cell specific genes. Total androgen receptor (total AR), androgen receptor splice variant 7 (AR-V7), and epithelial cell adhesion molecule (EpCAM) using primers and probes shown in Table 2. In brief, 20 µL ddPCR reactions contained 10 µL ddPCR Supermix for Probes (No dUTP) (Bio-Rad, Hercules, CA, USA), 500 nM of each relevant primer and 250 nM probe Fluorescein (6-FAM) or HEX. Total AR and AR-V7 reactions were multiplexed as previously-described [7]. Droplets were generated with 70 µL oil using a QX200 droplet generator (Bio-Rad). Amplification was performed at 95 °C for 10 min, followed by 40 cycles of 94 °C for 30 s and 55 °C for 1 min using a C1000 Touch Thermo Cycler (Bio-Rad). After amplification, the droplets were read with a QX200 Droplet Reader (Bio-Rad) and analysed with QuantaSoft software v1.7.4. The fluorescence thresholds used were 6-FAM 2000 for AR-V7, HEX 2500 for total AR, and HEX 1500 for EpCAM. Readings with ≥5 droplets were considered positive. The total error calculated by the software was used as the 95% confidence intervals of ddPCR measurements.

### 4.7. Statistical Analysis

Analysis of cell and RNA recovery was performed by two-way Analysis of Variance (ANOVA), using GraphPad Prism software v6.07 (GraphPad Software Inc., San Diego, CA, USA).

## 5. Conclusions

While spiked cell recovery was not affected by the blood tube type even after 48 h of storage, tumour cell-specific RNA was undetectable by ddPCR in CTCs from stored blood samples containing preservatives, likely due to crosslinking effects suppressing RNA accessibility. Surprisingly, AR-V7 was readily detectable in patient CTCs enriched from common EDTA blood tubes after up to 48 h. Although BCTs have been thoroughly tested for circulating tumour nucleic acid detection [28,29,30,31,32,33], and some initial studies of blood storage in BCTs for tumour cell analysis were also encouraging when using image-based cell analysis involving fluorescent probing for proteins or nucleic acids [23], our data suggests that RNA extraction and downstream analysis by PCR-based methods is severely impeded by the preservatives.

## Figures and Tables

**Table 1:** Androgen receptor variant 7 (AR-V7) and total AR expression in prostate cancer patient blood samples.

Patient	Hormone Sensitivity Status ^1^	CTC Count/mL Blood	AR-V7 Copies/mL Blood				Total AR Copies/mL Blood			
		<4 h *	<4 h *	4 h	24 h	48 h	<4 h *	4 h	24 h	48 h
**1**	CRPC	9 *	2 *	7	5	3	96 *	155	49	84
**2**	CRPC	2 *	110 *	126	128	74	19,140 *	7963	6083	4191
**3**	CRPC	6 *	45 *	210	135	102	2610 *	11,392	5994	3848

^1^ CRPC = castrate resistant prostate cancer. Patients who had 7–9 month previously high AR-V7 levels detected [7] were chosen for this study (* for comparison previous data are presented; note in the previous study CTC isolation was performed using a different instrument (IsoFlux, Fluxion, San Francisco, CA, USA), CTC counts are normalized per mL blood). New AR-V7 and total AR expression data of the same patients are presented.

**Table 2:** Primers and probes.

Gene	Primers (5′→3′)	Probes (5′→3′)
**Total AR**	F: GGA ATT CCT GTG CAT GAA AGC R: CGA TCG AGT TCC TTG ATG TAG TTC	[HEX] CTT CAG CAT TAT TCC AGT G [BHQ1]
**AR-V7**	F: CGG AAA TGT TAT GAA GCA GGG ATG A R: CTG GTC ATT TTG AGA TGC TTG CAA T	[6FAM] TCT GGG AGA AAA ATT CCG [BHQ1]
**EpCAM**	F: CGT CAA TGC CAG TGT ACT TCA R: TTT CTG CCT TCA TCA CCA AA	[HEX] TAC TGT CAT TTG CTC AAA GC [BHQ1]

AR: androgen receptor; AR-V7: androgen receptor variant 7; EpCAM: epithelial cell adhesion molecule; 6FAM: Fluorescein; BHQ1:black hole quencher 1. F: Forward, R: Reverse.

## Supplementary Materials

Supplementary materials can be found at www.mdpi.com/1422-0067/18/5/1047/s1.

## References

[1] Caixeiro N.J., Kienzle N., Lim S.H., Spring K.J., Tognela A., Scott K.F., Souza P.D., Becker T.M. (2014). Circulating tumour cells—A bona fide cause of metastatic cancer. Cancer Metastasis Rev..

[2] Joosse S.A., Gorges T.M., Pantel K. (2015). Biology, detection, and clinical implications of circulating tumor cells. EMBO Mol. Med..

[3] Becker T.M., Caixeiro N.J., Lim S.H., Tognela A., Kienzle N., Scott K.F., Spring K.J., de Souza P. (2014). New frontiers in circulating tumor cell analysis: A reference guide for biomolecular profiling toward translational clinical use. Int. J. Cancer.

[4] Krebs M.G., Metcalf R.L., Carter L., Brady G., Blackhall F.H., Dive C. (2014). Molecular analysis of circulating tumour cells—Biology and biomarkers. Nat. Rev. Clin. Oncol..

[5] De Bono J.S., Scher H.I., Montgomery R.B., Parker C., Miller M.C., Tissing H., Doyle G.V., Terstappen L.W.W.M., Pienta K.J., Raghavan D. (2008). Circulating tumor cells predict survival benefit from treatment in metastatic castration-resistant prostate cancer. Clin. Cancer Res..

[6] Antonarakis E.S., Lu C., Luber B., Wang H., Chen Y., Nakazawa M., Nadal R., Paller C.J., Denmeade S.R., Carducci M.A. (2015). Androgen receptor splice variant 7 and efficacy of taxane chemotherapy in patients with metastatic castration-resistant prostate cancer. JAMA Oncol..

[7] Ma Y., Luk A., Young F.P., Lynch D., Chua W., Balakrishnar B., de Souza P., Becker T.M. (2016). Droplet digital PCR based androgen receptor variant 7 (AR-V7) detection from prostate cancer patient blood biopsies. Int. J. Mol. Sci..

[8] Antonarakis E.S., Lu C., Luber B., Wang H., Chen Y., Zhu Y., Silberstein J.L., Taylor M.N., Maughan B.L., Denmeade S.R. (2017). Clinical significance of androgen receptor splice variant-7 mRNA detection in circulating tumor cells of men with metastatic castration-resistant prostate cancer treated with first- and second-line abiraterone and enzalutamide. J. Clin. Oncol..

[9] Liotta L.A., Kleinerman J., Saidel G.M. (1974). Quantitative relationships of intravascular tumor cells, tumor vessels, and pulmonary metastases following tumor implantation. Cancer Res..

[10] Butler T.P., Gullino P.M. (1975). Quantitation of cell shedding into efferent blood of mammary adenocarcinoma. Cancer Res..

[11] Chang Y.S., Tomaso E.D., McDonald D.M., Jones R., Jain R.K., Munn L.L. (2000). Mosaic blood vessels in tumors: Frequency of cancer cells in contact with flowing blood. Proc. Natl. Acad. Sci. USA.

[12] Meng S., Tripathy D., Frenkel E.P., Shete S., Naftalis E.Z., Huth J.F., Beitsch P.D., Leitch M., Hoover S., Euhus D. (2004). Circulating tumor cells in patients with breast cancer dormancy. Clin. Cancer Res..

[13] Sun Y., Yang X., Zhou J., Qiu S., Fan J., Xu Y. (2011). Circulating tumor cells: Advances in detection methods, biological issues, and clinical relevance. J. Cancer Res. Clin. Oncol..

[14] Yu M., Stott S., Toner M., Maheswaran S., Haber D.A. (2011). Circulating tumor cells: Approaches to isolation and characterization. J. Cell Biol..

[15] Alix-Panabières C., Pantel K. (2014). Challenges in circulating tumour cell research. Nat. Rev. Cancer.

[16] Lam N.Y.L., Rainer T.H., Chiu R.W.K., Lo Y.M.D. (2004). EDTA is a better anticoagulant than heparin or citrate for delayed blood processing for plasma DNA analysis. Clin. Chem..

[17] Palmirotta R., Ludovici G., de Marchis M.L., Savonarola A., Leone B., Spila A., de Angelis F., Morte D.D., Ferroni P., Guadagni F. (2011). Preanalytical procedures for DNA studies: The experience of the interinstitutional multidisciplinary BioBank (BioBIM). Biopreserv. Biobank..

[18] Fehm T., Solomayer E.F., Meng S., Tucker T., Lane N., Wang J., Gebauer G. (2005). Methods for isolating circulating epithelial cells and criteria for their classification as carcinoma cells. Cytotherapy.

[19] Fernando M.R., Chen K., Norton S., Krzyzanowski G., Bourne D., Hunsley B., Ryan W.L., Bassett C. (2010). A new methodology to preserve the original proportion and integrity of cell-free fetal DNA in maternal plasma during sample processing and storage. Prenat. Diagn..

[20] Fernando M.R., Norton S.E., Luna K.K., Lechner J.M., Qin J. (2012). Stabilization of cell-free RNA in blood samples using a new collection device. Clin. Biochem..

[21] Warrino D.E., DeGennaro L.J., Hanson M., Swindells S., Pirruccello S.J., Ryan W.L. (2005). Stabilization of white blood cells and immunologic markers for extended analysis using flow cytometry. J. Immunol. Methods.

[22] Yee S.S., Lieberman D.B., Blanchard T., Rader J., Zhao J., Troxel A.B., DeSloover D., Fox A.J., Daber R.D., Kakrecha B. (2016). A Novel approach for next-generation sequencing of circulating tumor cells. Mol. Genet. Genom. Med..

[23] Qin J., Alt J.R., Hunsley B.A., Williams T.L., Fernando M.R. (2014). Stabilization of circulating tumor cells in blood using a collection device with a preservative reagent. Cancer Cell Int..

[24] Qin J., Williams T.L., Fernando M.R. (2013). A novel blood collection device stabilizes cell-free RNA in blood during sample shipping and storage. BMC Res. Notes.

[25] Das K., Norton S.E., Alt J.R., Krzyzanowski G.D., Williams T.L., Fernando M.R. (2014). Stabilization of cellular RNA in blood during storage at room temperature: A comparison of cell-free RNA BCT with K3EDTA tubes. Mol. Diagn. Ther..

[26] Flores L.M., Kindelberger D.W., Ligon A.H., Capelletti M., Fiorentino M., Loda M., Cibas E.S., Jänne P.A., Krop I.E. (2010). Improving the yield of circulating tumour cells facilitates molecular characterisation and recognition of discordant HER2 amplification in breast cancer. Br. J. Cancer.

[27] Tsui N.B.Y., Ng E.K.O., Lo Y.M.D. (2002). Stability of endogenous and added RNA in blood specimens, serum, and plasma. Clin. Chem..

[28] Denis M.G., Knol A., Théoleyre S., Vallée A., Dréno B. (2015). Efficient detection of BRAF mutation in plasma of patients after long-term storage of blood in cell-free DNA blood collection tubes. Clin. Chem..

[29] Schiavon G., Hrebien S., Garcia-Murillas I., Cutts R.J., Pearson A., Tarazona N., Fenwick K., Kozarewa I., Lopez-Knowles E., Ribas R. (2015). Analysis of ESR1 mutation in circulating tumor DNA demonstrates evolution during therapy for metastatic breast cancer. Sci. Transl. Med..

[30] Toro P.V., Erlanger B., Beaver J.A., Cochran R.L., VanDenBerg D.A., Yakim E., Cravero K., Chu D., Zabransky D.J., Wong H.Y. (2015). Comparison of cell stabilizing blood collection tubes for circulating plasma tumor DNA. Clin. Biochem..

[31] Diaz I.M., Nocon A., Mehnert D.H., Fredebohm J., Diehl F., Holtrup F. (2016). Performance of streck cfDNA blood collection tubes for liquid biopsy testing. PLoS ONE.

[32] Kang Q., Henry N.L., Paoletti C., Jiang H., Vats P., Chinnaiyan A.M., Hayes D.F., Merajver S.D., Rae J.M., Tewari M. (2016). Comparative analysis of circulating tumor DNA stability in K3EDTA, Streck, and CellSave blood collection tubes. Clin. Biochem..

[33] Sherwood J.L., Corcoran C., Brown H., Sharpe A.D., Musilova M., Kohlmann A. (2016). Optimised pre-analytical methods improve KRAS mutation detection in circulating tumour DNA (ctDNA) from patients with non-small cell lung cancer (NSCLC). PLoS ONE.

