# Supplementary figures and images for: CTC-mRNA (AR-V7) Analysis from Blood Samples—Impact of Blood Collection Tube and Storage Time
Source: Int J Mol Sci. 2017 May 12;18(5):1047. doi: 10.3390/ijms18051047 (PMC5454959; doi:10.3390/ijms18051047)

Spiked samples

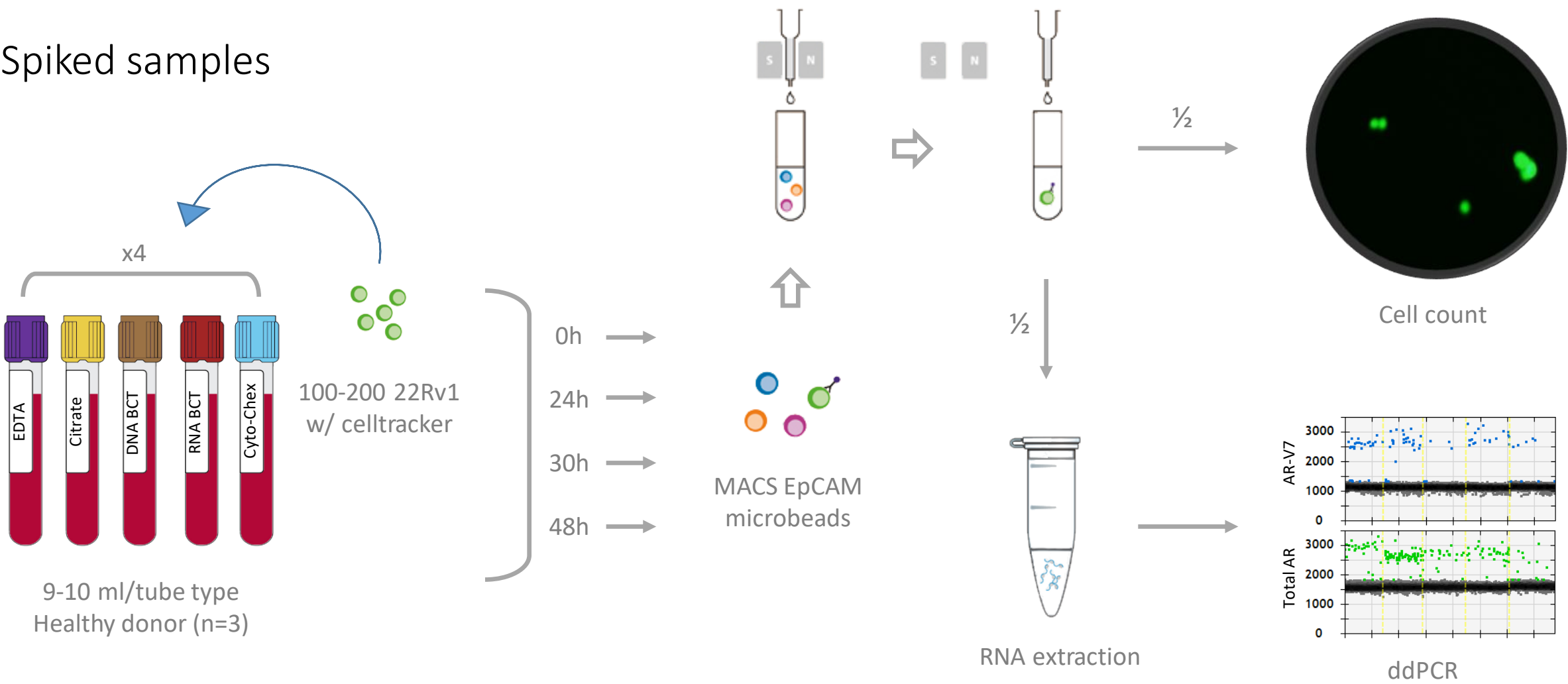

Increased Proteinase K treatment

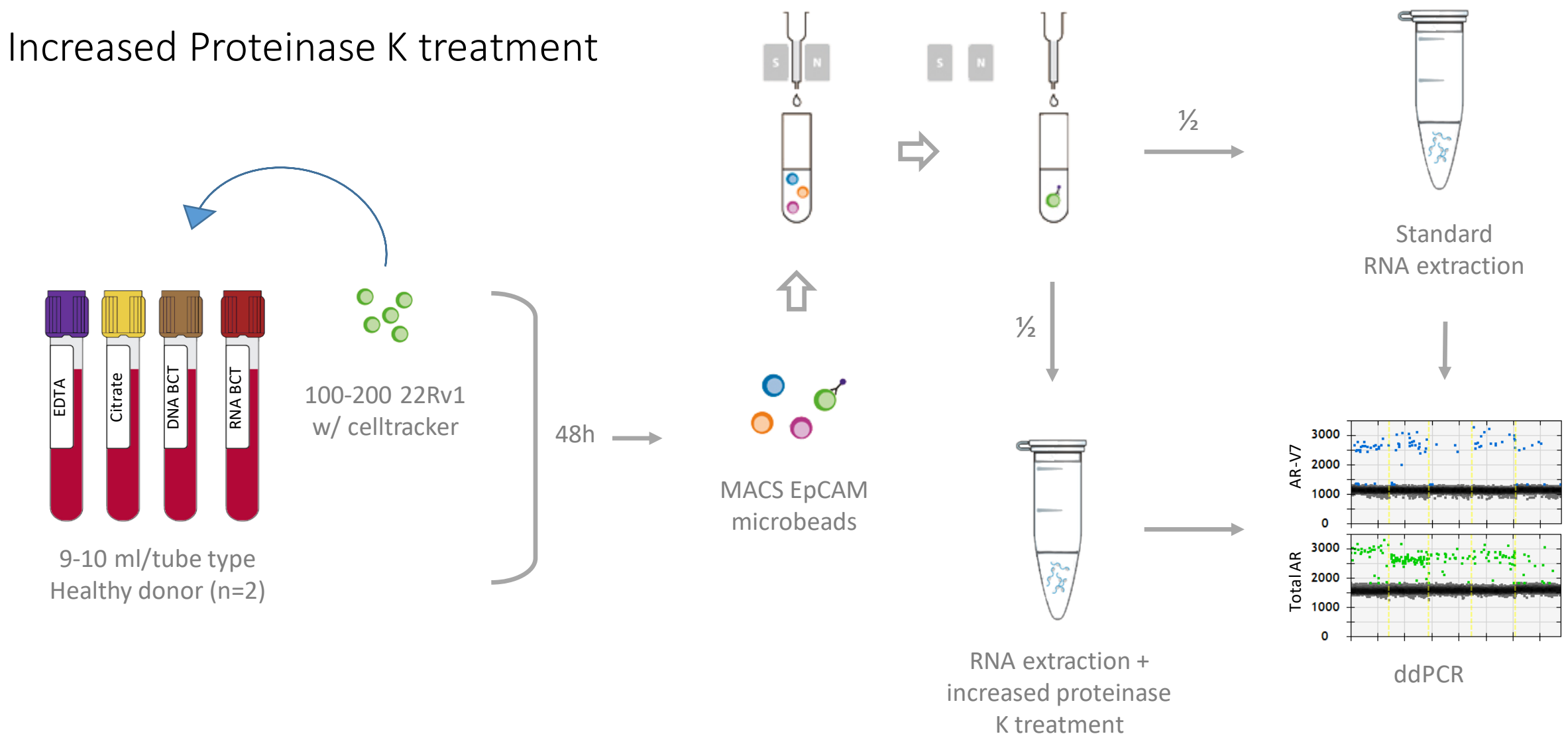

Patient samples

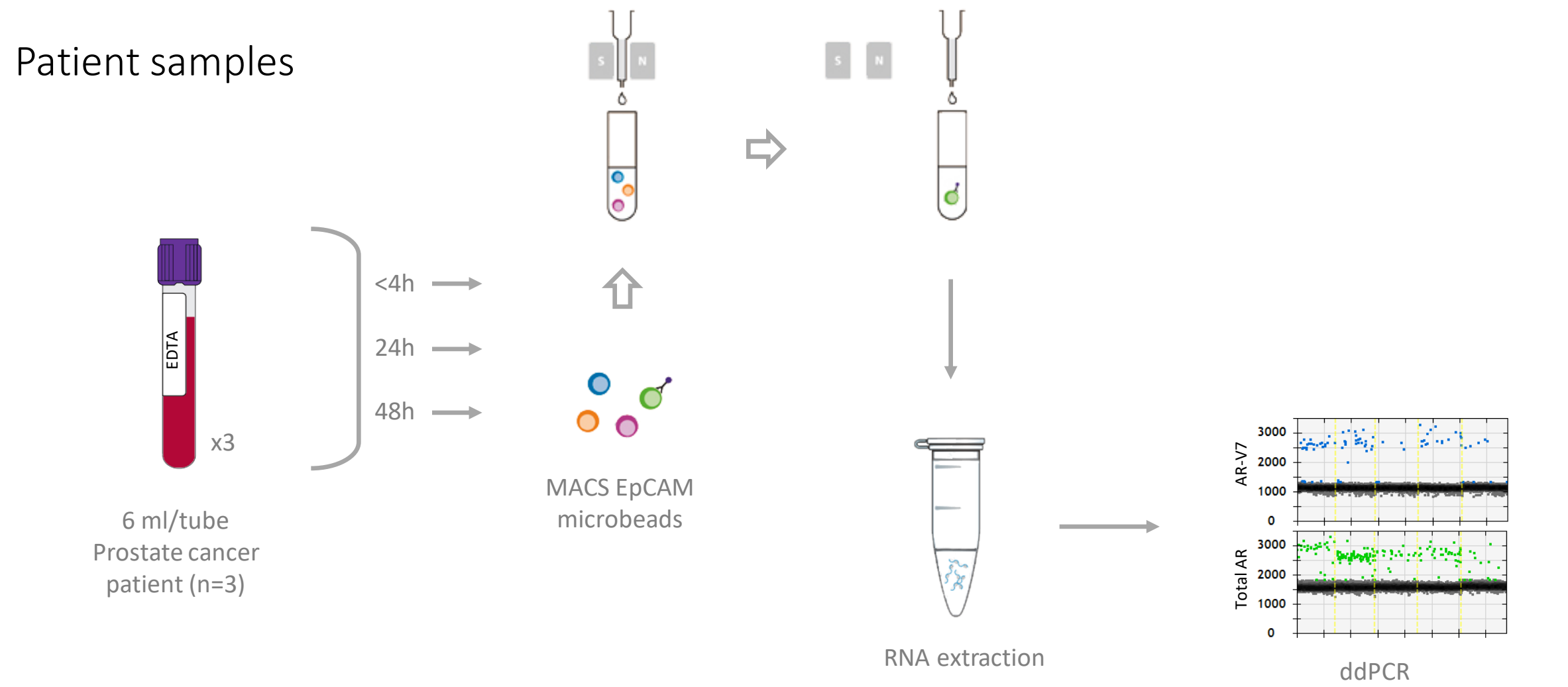

Supplement: Supplementary file 1 [file ijms-18-01047-s001.pdf]
